# Grapevine Grafting: Scion Transcript Profiling and Defense-Related Metabolites Induced by Rootstocks

**DOI:** 10.3389/fpls.2017.00654

**Published:** 2017-04-27

**Authors:** Walter Chitarra, Irene Perrone, Carla G. Avanzato, Andrea Minio, Paolo Boccacci, Deborah Santini, Giovanna Gilardi, Ilenia Siciliano, Maria L. Gullino, Massimo Delledonne, Franco Mannini, Giorgio Gambino

**Affiliations:** ^1^Institute for Sustainable Plant Protection, National Research CouncilTorino, Italy; ^2^Council for Agricultural Research and Economics, Viticulture Research CenterConegliano, Italy; ^3^Department of Biotechnology, University of VeronaVerona, Italy; ^4^AGROINNOVA, Centre for Innovation in the Agro-Environmental Sector, University of TorinoGrugliasco, Italy

**Keywords:** *Vitis*, abscisic acid, stilbene compounds, transcriptome, pathogens, *Plasmopara viticola*

## Abstract

Rootstocks are among the main factors that influence grape development as well as fruit and wine composition. In this work, rootstock/scion interactions were studied using transcriptomic and metabolic approaches on leaves of the “Gaglioppo” variety, grafted onto 13 different rootstocks growing in the same vineyard. The whole leaf transcriptome of “Gaglioppo” grafted onto five selected rootstocks showed high variability in gene expression. In particular, significant modulation of transcripts linked to primary and secondary metabolism was observed. Interestingly, genes and metabolites involved in defense responses (e.g., stilbenes and defense genes) were strongly activated particularly in the GAG-41B combination, characterized in addition by the down-regulation of abscisic acid (ABA) metabolism. On the contrary, the leaves of “Gaglioppo” grafted onto 1103 Paulsen showed an opposite regulations of those transcripts and metabolites, together with the greater sensitivity to downy mildew in a preliminary *in vitro* assay. This study carried out an extensive transcriptomic analysis of rootstock effects on scion leaves, helping to unravel this complex interaction, and suggesting an interesting correlation among constitutive stilbenes, ABA compound, and disease susceptibility to a fungal pathogen.

## Introduction

Grapevine yield and quality depend on several factors, such as genotype, biotic, and abiotic stresses, agro-technical practices, all interacting with each other and influencing development as well as fruit and wine quality (Marè et al., 2013). The relationship between scion and environment is also mediated by the rootstock, obtained by crossing a large variety of *Vitis* species, such as *Vitis berlandieri, Vitis riparia*, and *Vitis rupestris*. In general, grafting influences vigor, yield, water, nutrient, and hormone metabolism, thereby impacting the function of the whole plant and its responses to stress events. In particular, the long distance transport of mRNAs, proteins, and small RNAs contributes to explain the molecular mechanism for how graft partners interact with each other to produce a successful vine graft. Interestingly, genotypes, and scion/rootstock combinations are among the factors influencing the extent of mRNA exchange across the graft junction, as well as the species of mobile mRNAs and the direction of mRNA movement (Yang et al., 2015; Warschefsky et al., 2016; Pagliarani et al., 2017).

Phylloxera-resistant rootstocks have been required in Europe since the 19th century and represent the most prolonged use of a biological control strategy against a pest. Since the last decade, research on this topic has aimed to develop more efficient and sustainable practices providing resistance to other pests and ameliorating environmental adaptation (Anwar et al., 2002). Besides the physical and chemical mechanisms provided by tolerant rootstocks to limit the pathogen progression, not much is known about the ability of the rootstock to provide increased tolerance against scion diseases also through molecular mechanisms. Apple cultivars susceptible to fire blight disease are usually grafted to resistant rootstocks, and it has been interestingly observed in field a different degree of resistance depending on the rootstock employed (Cline et al., 2001).

The common commercial rootstocks used in viticulture and their phenotypes have been characterized (Tramontini et al., 2013; Corso and Bonghi, 2014). Studies investigating the molecular network of rootstock and scion interactions are still scarce, although recently developed “-omics” tools have partially unraveled complex rootstock/scion interactions. Marguerit et al. (2012), for example, dissected the genetic architecture of scion transpiration control and its acclimation to drought using QTL detection traits, defining the rootstock genetic control of these processes. In another study, Marè et al. (2013) showed the effects of different soil compositions and rootstocks on leaf transcriptomic modulation of Pinot Noir. Similarly, Corso et al. (2015) used a comparative transcriptome approach on drought tolerant and susceptible rootstock genotypes in response to water stress. The authors showed variations in modulation of genes involved in the phenylpropanoid pathway (mainly stilbene and flavonoid genes), suggesting a resveratrol-mediated way to enhance water stress resilience in a tolerant rootstock genotype. Stilbenoids (resveratrol and viniferin in particular) have reactive oxygen species (ROS) scavenging activity and act as a powerful defense system against biotic stresses (e.g., powdery mildew, downy mildew; Dabauza et al., 2015; Houillé et al., 2015). Furthermore, it is well-known that *Vitis vinifera* represents the best nutritional source of stilbenoid compounds with valuable effects on human health; these compounds have numerous pharmacological activities including anti-fungal, anti-inflammatory and anti-tumor effects (Bai et al., 2010; Zhang et al., 2010; Houillé et al., 2014). Constitutive stilbene production in plants and accumulation in the final product (berries and wines) depends on grape variety, climate, soil, and other factors (Vincenzi et al., 2013). These and other secondary metabolites play a key role in grapevine constitutive and induced defense responses to biotic and/or abiotic stresses. Priming the defense state represents an important issue in grapevine immune system modulation that needs to be elucidated (Chong et al., 2009).

In this work, rootstock/scion interactions were studied using transcriptomic and biochemical approaches on leaves of Gaglioppo variety (Librandi CVT 80 clone), typically cultivated in Southern Italy, grafted onto 13 selected rootstocks grown under same environmental conditions. The goals of this study were therefore to analyze the transcript modulations induced in scion leaves in response to grafting onto different rootstocks, and to evaluate constitutive levels of stress, defense-related genes and metabolites induced by different rootstocks and involved in priming defense responses.

## Materials and methods

### Vineyard description and experimental set-up

The study was carried out during two consecutive seasons (2014 and 2015) in a vineyard located in the South of Italy at the Librandi Estate (Cirò Marina, Calabria region), characterized by clay-loam soil (pH 7.9, organic matter content 1.13%, cation exchange capacity, 30.92 meq/100 g soil) and climate conditions as reported in Supplementary Figure S1. Scions of “Gaglioppo” Librandi CVT 80 clone were grafted onto 13 different rootstocks as described in Table 1. All vines were 3 years old and planted at a density of 7,300 plants ha^−1^, with a spacing of 1.60 × 0.85 m. Plants were trained to a vertical trellis system with spur pruning; conventional agronomic management was regularly applied in the vineyard. Water was supplied by means of drip irrigation. Drippers were positioned around 50 cm from ground and 10 cm from vine trunk. During the vegetative season, each vine was watered 5 times with 30 liters as follows: one in June, two in July and two in August. Rainfall events were recorded as shown in the Supplementary Figure S1. The 13 graft combinations were located in adjacent blocks in the vineyard, and for each of them, nine plants were considered. For each plant, two fully expanded leaves inserted in the central region of the shoot and of approximately equivalent physiological stage and condition, were collected at the end of August 2014. Leaves from three plants of each combination were pooled to form a biological replicate (2 leaves × 3 plants). Three biological replicates were carried out and immediately frozen in liquid nitrogen. Samples were stored at −80°C until molecular and biochemical analyses. Some agronomic parameters were determined for each vine, i.e., bunch weight and yield at harvest, and vigor measuring the pruning mass at winter time in 2 consecutive years (2014, 2015; see Table 1 reporting the average of the data collected in the 2014 and in the 2015 vintages).

**Table 1:** **Description of the graft combinations analyzed**.

**Rootstocks**	**Genetic origin**	**Short name**	**Bunch weight/plant (Kg)**	**Yield/plant (Kg)**	**Vigour pruned wood/plant (Kg)**
140 Ruggeri	*V. rupestris* × *V. berlandieri*	GAG-140R	0.26 ± 0.16a	2.12 ± 0.78ab	0.36 ± 0.19bc
**1103 Paulsen**		**GAG-1103P**	**0.39** ± **0.15b–d**	**3.45** ± **1.51cd**	**0.66** ± **0.18de**
**17–37 Mgt**		**GAG-17-37**	**0.40** ± **0.09cd**	**3.54** ± **0.68cd**	**0.41** ± **0.05b–d**
775 Paulsen		GAG-775P	0.41 ± 0.09cd	4.26 ± 1.96d	0.95 ± 0.33f
779 Paulsen		GAG-779P	0.38 ± 0.03b–d	2.87 ± 1.28a–d	0.44 ± 0.17c–e
110 Richter		GAG-110R	0.39 ± 0.08cd	1.92 ± 0.96ab	0.25 ± 0.06a–c
420 A	*V. riparia* × *V. berlandieri*	GAG-420A	0.30 ± 0.09a–c	2.11 ± 0.86ab	0.23 ± 0.10ab
SO4		GAG-SO4	0.49 ± 0.11de	3.69 ± 1.05cd	0.82 ± 0.32f
**Kober 5 BB**		**GAG-KOB**	**0.31** ± **0.13a–c**	**2.34** ± **1.16a–c**	**0.43** ± **0.11b–e**
Gravesac	161-49C (*V. riparia* × *V. berlandieri*) × 3309 C	GAG-GRAV	0.36 ± 0.09b–d	3.52 ± 0.98cd	0.65 ± 0.14de
**3309 Couderc**	*V. riparia* × *V. rupestris*	**GAG-3309C**	**0.41** ± **0.09cd**	**3.28** ± **0.91b–d**	**0.41** ± **0.12b–e**
Rupestris du Lot	*V. rupestris*	GAG-RUP	0.57 ± 0.11e	3.55 ± 1.13cd	0.68 ± 0.13e
**41 B Mgt**	*V. vinifera* (Chasselas) x *V. berlandieri*	**GAG-41B**	**0.26** ± **0.0ab**	**1.45** ± **0.74a**	**0.13** ± **0.04a**

*The scion Vitis vinifera cv. “Gaglioppo,” Librandi CVT80 clone (GAG-) was grafted onto 13 different rootstocks. The combinations chosen for RNA-seq analyses are indicated in bold font. Grape weight and yield data collected at harvest and vine vigor are reported as average of the data collected in the 2014 and in the 2015 vintages. Letters indicating significant differences by Tukey's HSD-test (P < 0.05) are reported for each value. Data are expressed as mean ± SD (n = 3)*.

### RNA sequencing analyses

Total RNA was extracted using the Spectrum™ Plant Total RNA extraction kit (Sigma Aldrich) starting from 100 mg of material, and RNA quantity was checked using a NanoDrop 1000 spectrophotometer (Thermo Fisher Scientific). RNA sample quality was checked on an RNA 6000 Nano Labchip using a Bioanalyzer 1000 (Agilent Technologies, Santa Clara, USA); all samples were RIN ≥ 7. cDNA libraries were prepared using TruSeq RNA Sample Prep Kit v2 (Illumina, San Diego, USA), starting from 2.5 μg of total RNA. Selected mRNAs were sheared for 8 min and finished libraries were amplified using 12 cycles of PCR. Libraries were validated on a DNA 1000 Chip using a Bioanalyzer 1000 (Agilent Technologies, Santa Clara, USA) and then quantified through qPCR using KAPA SYBR FAST Universal qPCR kit (Kapa Biosystems, Wilmington, USA) and an internal standard curve. Finally, a 100 SR sequencing run was performed using TruSeq SBS Kit v3-HS and TruSeq PE Cluster Kit v3-cBot-HS kits on a HiSeq 1000 system (Illumina, San Diego, USA).

### Reads pre-processing, alignment, and expression analysis

Sequenced reads underwent quality filtering prior to expression analysis. Low-quality reads (>50 bases with quality < 7 or >10% undetermined bases) were removed with a custom script, sequencing adapter sequences were clipped using Scythe (https://github.com/vsbuffalo/scythe), and low-quality bases at the 3′ ends of reads were trimmed using a quality threshold of 20 over a 10-base window with Sickle (https://github.com/najoshi/sickle) discarding reads shorter than 20 bp.

Reads were aligned against the PN40024 genome sequence using TopHat v2.0.14 (Trapnell et al., 2012), running Bowtie2 ver.2.2.4 (Siragusa et al., 2013) in very sensitive mode (parameter: –b2-very-sensitive) with IGGP_12x ver. 16 as the reference annotation. Cufflinks ver.2.2.0 (Trapnell et al., 2012) was used to calculate the expression values of all known genes in FPKM (fragments per kilobase of exon per million fragments mapped) using a geometric method for the normalization and setting a False Discovery Rate (FDR) ≤ 5% for the detection of significantly differentially expressed genes among the conditions. Expression values and next-generation sequencing data are available in the Gene Expression Omnibus (GEO) public database with accession number GSE82317. The identification of differentially expressed genes (DEGs) was performed using DESeq software (Zenoni et al., 2010).

Hierarchical clustering (HCL) analysis was applied using Pearson's correlation distance and MeV v4.9 software (http://www.tm4.org/mev.html) using as input log2 transformed FPKM values of genes in the five graft combinations.

All transcripts were annotated against the V1 version of the 12X draft annotation of the grapevine genome (http://genomes.cribi.unipd.it/DATA/). Blast2GO (Conesa et al., 2005) software was used to assign gene ontology (GO) terms, and transcripts were grouped into functional categories based on GO biological processes (GO:0005975, Carbohydrate Metabolism; GO:0044036, Cell Wall Metabolism; GO:0006520, Amino Acid and Derivative Metabolism; GO:0019725, Cellular Homeostasis; GO:0009987, Cellular Processes; GO:0032502, Developmental Process; GO:0090304, DNA/RNA Metabolism; GO:0006091, Energy Pathways; GO:0006629, Lipid Metabolism; GO:0009725, Response to Hormone; GO:0006950, Response to Stress; GO:0019748, Secondary Metabolism; GO:0007165, Signal Transduction; GO:0051090, Transcription Factor Activity Regulation; GO:0006810, Transport). Genes with unknown functions, with “No Hit” annotations and encoding pentatricopeptide (PPR) repeat-containing proteins were also included.

Metabolic pathways were visualized using MapMan software Version 3.6.0RC1 (Thimm et al., 2004; http://mapman.gabipd.org/web/guest/mapman), integrated for grapevine with information from Nimblegen and Affymetrix platforms, as previously reported (Rotter et al., 2009; Dal Santo et al., 2013).

### RT-qPCR analysis of gene expression

Total RNA was treated with DNase I (Invitrogen, Thermo Fisher Scientific) in accordance with the manufacturer's instructions. For each biological replicate, first-strand cDNA was synthesized starting from 2 μg of total RNA using the High Capacity cDNA Reverse Transcription kit (Applied Biosystems, Thermo Fisher Scientific) according to the manufacturer's instructions. Gene-specific primers (Supplementary Table S5) were designed using Primer Express® software (v3.0, Applied Biosystems, Thermo Fisher Scientific). Reactions were carried out using Power SYBR® Green PCR Master Mix (Applied Biosystems, Thermo Fisher Scientific) as reported in Gambino et al. (2012). Three technical replicates were run for each biological replicate, and the expression of transcripts was quantified after normalization to two housekeeping genes: ubiquitin (*VvUBI*) and actin1 (*VvACT1*). The results were calculated as expression ratios (relative quantity, RQ) to GAG-1103P.

### HPLC-MS/MS analysis of stilbenes and abscisic acid (ABA) content

Stilbenes and ABA were quantified starting from 200 mg of frozen leaves. Reversed-phase high-performance liquid chromatography analysis was performed on a 1,260 Agilent Technologies system. Chromatographic separation was performed with a Phenomenex (Torrance, CA) Luna C18 column (150 × 2.1 mm, 3 μm particle size), with a C18 SecurityGuard column (4.0 × 3.0 mm ID), operated at room temperature. Elution was carried out using aqueous formic acid (0.1% v/v; mobile phase A) and acetonitrile (mobile phase B). HPLC analysis was performed using a gradient from 20 to 60% of mobile phase B in 15 min, then from 60 to 100% of B in 4 min; after washing for 5 min with solvent B, the column was re-equilibrated. The flow rate was 0.2 mL/min and injection volume was 10 μL. A triple quadrupole mass spectrometer (Varian 310-MS TQ Mass Spectrometer) equipped with an ESI interface operated in negative ion mode was used. The detection of analytes was carried out in multiple reaction monitoring (MRM) mode by monitoring two transitions for each compound. The concentration of analytes was quantified using an external calibration method. Original standards of resveratrol (purity ≥99%), polydatin (purity ≥95%), and viniferin (purity ≥95%), purchased from Sigma-Aldrich, were used to prepare the calibration curves. Similarly, the ABA content was quantified as described by Siciliano et al. (2015).

### *In vitro* downy mildew pathogenicity test

Leaf disks were cut with a cork borer (9 mm in diameter) from leaves collected in August from each selected scion/rootstock combination. Ten leaf disks were placed with the abaxial surface up on water-saturated filter paper sheets (Whatman®, Sigma-Aldrich) in 90 mm Petri plates. Leaf disks from the same rootstock/scion combination inoculated with sterile water served as control.

The inoculum was prepared by rinsing leaves infected with the AG1 *Plasmopara viticola* population from a greenhouse-grown susceptible selection of grape cv. Chardonnay with distilled water and adjusting to 5 × 10^4^ sporangia/mL using a hemocytometer. The inoculum was then pipetted onto the abaxial surface of each leaf disk using 30 μL/leaf disk. Petri plates were placed in a growth chamber at 22 ± 1°C under a 16 h photoperiod. The experimental design was a randomized complete block with four replications of six leaf disks each with a total of 18 points of inoculation.

Leaf disks were visually examined 7–10 days after inoculation for sporulation using a scale from 0 to 3: 0 = no symptoms; 1 = >0 to 25%; 2 = >26 to 50%; 3 = >50% of the affected area developed from the drop. Disease severity was estimated on 24 disks leaf/cultivar.

### Statistical analyses

Data management and calculations were performed using a Microsoft Excel spreadsheet. One-way analyses of variance (ANOVA) with treatment as the main factor were performed with the SPSS 23.0 statistical software package (SPSS Inc., Cary, NC, USA). Tukey's HSD-test was applied when ANOVA showed significant differences (*P* < *0.05*). The standard deviation (SD) or the standard error (SE) of all means were calculated.

## Results and discussion

### The rootstock influences the agronomic traits of the scion

Agronomic, metabolic, and molecular changes induced on *V. vinifera* cv. Gaglioppo by different rootstocks were investigated in a vineyard located in the Calabria region (Southern Italy), characterized by a Mediterranean climate, as confirmed by the meteorological data collected on the experimental site in the summer 2014 (Supplementary Figure S1). These are the required conditions for the production of “Cirò,” the wine typical of the region derived from “Gaglioppo.”

In terms of agronomic traits, the results collected during the 2014 and 2015 vintages confirmed that the rootstock has a significant influence on yield and vigor of the scion (Table 1). In particular, the GAG-41B combination was characterized by very low agronomical performance in comparison with other rootstocks that induce high vigor and yield in “Gaglioppo” (e.g., 1103 Paulsen, 17–37 Mgt, 775 Paulsen, Gravesac, Rupestris du Lot and SO4). Taking into account those results (Table 1) and considering the genetic origin of rootstocks, that provide a good source of variability, five graft combinations were selected for further transcriptomic analyses by RNA-Seq. In more detail, among the rootstocks originated by the cross *V. rupestris* × *V. berlandieri*, we choosed the 1103 Paulsen because is extensively used in all regions characterized by Mediterranean climate for its high vigor and good tolerance to drought (Lovisolo et al., 2016). It could be considered as a reference rootstock for these viticultural regions and according to that, 1103 P was used as reference rootstock in our analyses. Among the crosses *V. rupestris* × *V. berlandieri*, we selected also the 17–37 Mgt since is extensively used in the Calabria region. Kober 5BB and 3309C induced medium vigor and yield in “Gaglioppo” (Table 1). In addition, 3309C is the only cross *V. riparia* × *V. rupestris* available in our vineyard, and the Kober 5BB is one of the more extensively used rootstock in Italy obtained by crossing of *V. riparia* × *V. berlandieri* (Table 1). Differently from any other combination, GAG-41B was characterized by lower agronomical performance and it is the only hybrid rootstock in our selection produced from a *V. vinifera* (“Chasselas”) parental.

### Rootstock affects the whole-transcriptome modulation in scion leaves

RNA-Seq produced 499.5 million fragments, equivalent to 49 Gb of total sequencing data with an average of 33 million fragments per sample (Supplementary Table S1). The sequences were quality filtered and aligned to PN40024 reference genome (IGGP 12X v16 assembly) with a mean success rate of 88%. Out of 29,970 annotated genes, 22,292 (74.4%) were found to be expressed (FPKM ≥ 0.1) and 17,050 were significantly differentially expressed genes (DEGs) in at least one rootstock/scion combination with respect to GAG-1103P, chosen as reference (*p*-value adjusted with Benjamin-Hochberg ≤ 0.5%; Supplementary Table S2). We decided then to apply a fold-change (FC) cut-off and to analyze only the genes whose expression was |log2FC| ≥ 2. We obtained 2,692 DEGs (Supplementary Table S3), the majority of them up-regulated (65%), meaning that these genes are over-expressed in the four graft combinations with respect to GAG-1103P.

**Figure 1:**
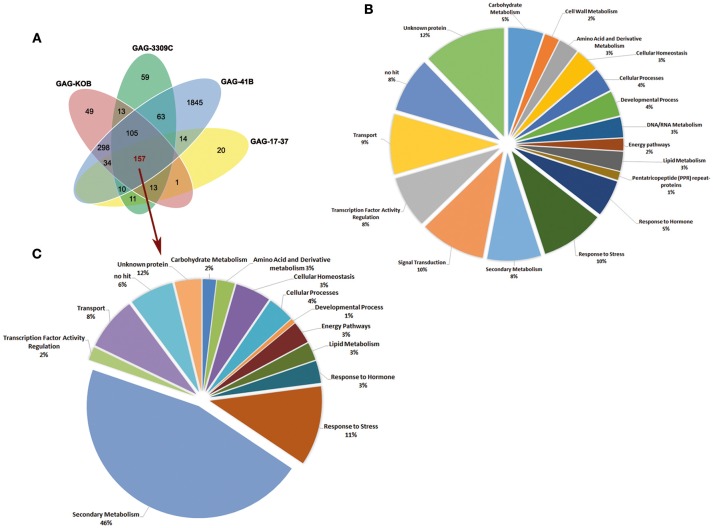
**Venn diagrams showing the distribution of the 2,692 DEGs (***P*** < 0.05; |log2FC| ≥ 2) in the graft combinations GAG-KOB, GAG-3309C, GAG-41B, and GAG-17-37, each compared to the reference GAG-1103P (A)**. Functional category distribution of the 2,692 DEGs **(B)** and of the 157 genes showing common differential expression in all the graft combinations **(C)**. Transcripts were grouped into the most represented functional categories, based on the GO classification of biological processes.

As shown by the Venn diagram in Figure 1A, the GAG-41B vs. GAG-1103P comparison showed the highest number of DEGs (2,526, around 94% of DEGs detected) and, interestingly, 1,845 (68%) were exclusively modulated in GAG-41B. On the contrary, only 260 genes showed different expression levels in GAG-17-37 with respect to the reference GAG-1103P, and 20 (0.7%) were genes exclusively affected in GAG-17-37. These numbers clearly suggest that 41 B Mgt rootstock induced very different responses in “Gaglioppo” at the molecular level with respect to 1103 Paulsen, whereas the scion showed similar transcriptomic changes when grafted onto 17–37 Mgt and 1103 Paulsen.

The 2,692 DEGs analyzed were annotated according to Grimplet et al. (2012), associated with their respective GO terms and then grouped in 18 highly-represented categories, based on GO biological processes (Figure 1B). The analysis of transcript functional categories revealed that the majority of DEGs are involved in stress responses, signal transduction processes, secondary metabolism, transport and transcription factor activity. Considering the 157 DEGs in common to all four graft combinations compared to reference GAG-1103P (Figure 1A), they were almost exclusively up-regulated (97%) and mainly involved in secondary metabolism (46%), with strong up-regulation of genes related to phenylpropanoid biosynthesis (Figure 1C).

**Figure 2:**
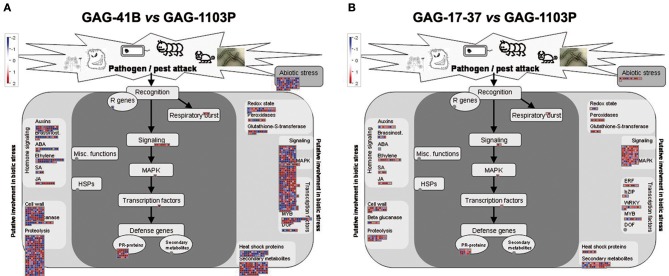
**MapMan software output to provide an overview of the effect of 41 B Mgt rootstock (A)** and 17–37 Mgt rootstock **(B)** induced on biotic stress responses and, in particular, on secondary metabolism. Log_2_ fold changes values referred to GAG-1103P are represented. Up-regulated and down-regulated transcripts are shown in red and blue, respectively.

A metabolic overview was performed with MapMan software to visualize the metabolic pathways in which DEGs are involved (Supplementary Figure S2A). In the GAG-41B vs. GAG-1103P comparison, the DEGs were distributed among all the metabolic pathways, although mainly concentrated in secondary metabolism (upregulation of terpenes, flavonoids, and phenylpropanoid genes) and in the photosynthesis process, with several up-regulated genes belonging to the two photosystems (Figure 2, Supplementary Figure S2B). Moreover, several DEGs were involved in hormonal responses, cell wall metabolism, and kinase signaling cascades, particularly in the activity of transcription factors such as bZIP, WRKY and MYB; overall, these metabolic changes affect plant responses to abiotic/biotic stresses (Figure 2). On the contrary, considering the comparison GAG-17-37 vs. GAG-1103P, a small number of DEGs were involved in responses to stress and secondary metabolism, while there were no differentially expressed genes involved in photosynthetic pathways (Figure 2, Supplementary Figure S2B).

**Figure 3:**
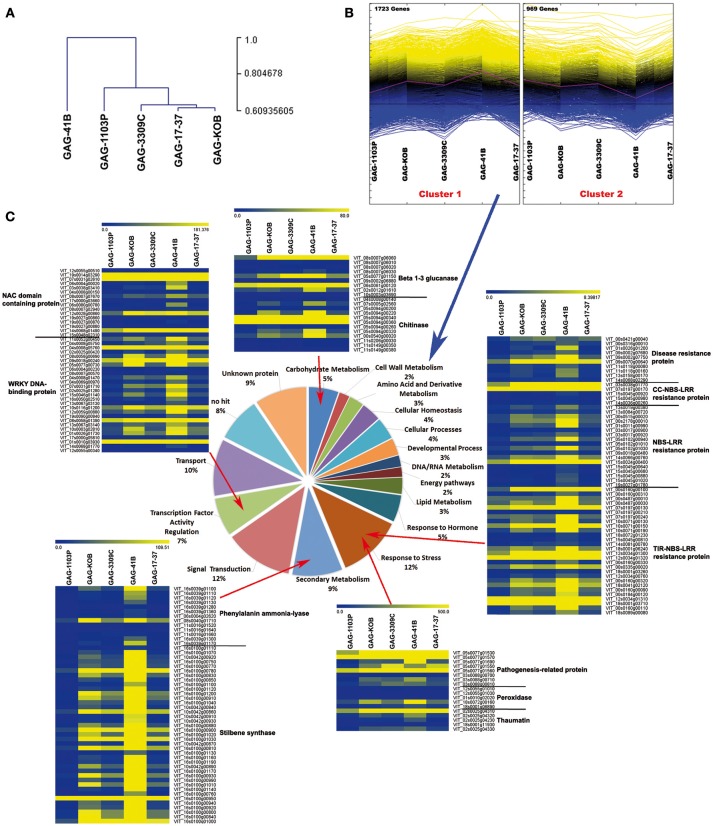
**Transcriptomic reprogramming induced in the scion “Gaglioppo” by different rootstocks**. Cluster dendrogram of GAG-41B, GAG-1103P, GAG-3309C, GAG-17-37, and GAG-KOB obtained using the average expression value of the three biological replicates **(A)**. Hierarchical clustering analysis of transcripts that were differentially modulated among the five different graft combinations (*P* < 0.05; |log2FC| ≥ 2) **(B)**. Functional category distribution of the transcripts belonging to Cluster 1, grouped into the most represented functional categories, based on the GO classification of biological processes **(C)**. The heat maps of transcriptional profiles associated with the categories “Carbohydrate metabolism,” “Response to stress,” “Secondary metabolism,” and “Transcription factor activity regulation” were generated with TMeV 4.9 using the average expression value of the three biological replicates.

In order to further analyze the data, a hierarchical clustering (HCL) analysis involving the DEGs with |log2FC| ≥2 was conducted to investigate the relationships of similarity among the five graft combinations. GAG-41B formed a completely separate clade from the others, whereas the remaining rootstock/scion combinations clustered together in a second group, in agreement with the fact that 41 B Mgt rootstock induced the most different transcriptomic changes in “Gaglioppo” (Figure 3A). In addition, the 2,692 DEGs clustered in two major groups (Figure 3B): Cluster 1 is characterized by 1,723 genes up-regulated in GAG-41B, partially up-regulated in GAG-KOB and less expressed in GAG-1103P, whereas Cluster 2 includes the remaining 969 genes down-regulated in GAG-41B with respect to the other combinations (Supplementary Table S4).

In Cluster 1, the carbohydrate metabolism category is highly represented by genes such as *beta-glucanases*, involved in callose degradation, and *chitinases*. Similarly, other genes related to biotic stress responses such as disease resistance proteins (NBS and NBS-LRR class), pathogenesis-related proteins as well as genes involved in jasmonate metabolism, lipid metabolism, phenylpropanoid metabolism, NAC domain-containing proteins, and WRKY DNA-binding proteins were induced in “Gaglioppo,” in particular by the 41 B Mgt rootstock (Cluster 1; Supplementary Table S4, Figure 3C). Interestingly, genes involved in the ABA metabolism were instead up-regulated in GAG-1103P (Cluster 2; Supplementary Table S4).

### A glimpse on the defense-related responses induced by the rootstock

Priming plant defense has obtained increasing attention in the last decade, and several studies have been conducted to enhance multiple stress tolerance by biological, genetic, or chemical methodologies (Savvides et al., 2016). Defense priming is defined as an adaptive, low-cost defensive measure in which the plant is in a persistently primed state of enhanced defense readiness (Martinez-Medina et al., 2016). The priming stimulus leading to this state can be of different nature and studies have revealed the usual presence of defense priming in field conditions (Heil and Silva Bueno, 2007). Our results show how rootstocks are potentially involved in the priming phenomenon, influencing the response of the scion to the environment through different strategies, with the 1103 Paulsen and 41 B Mgt rootstocks representing two opposite examples regarding the regulation of scion metabolism.

#### Phenylpropanoid metabolism

Stilbenoids show ROS scavenging activity acting as a powerful defense system against biotic stresses (e.g., powdery mildew, downy mildew; Dabauza et al., 2015; Houillé et al., 2015).

**Figure 4:**
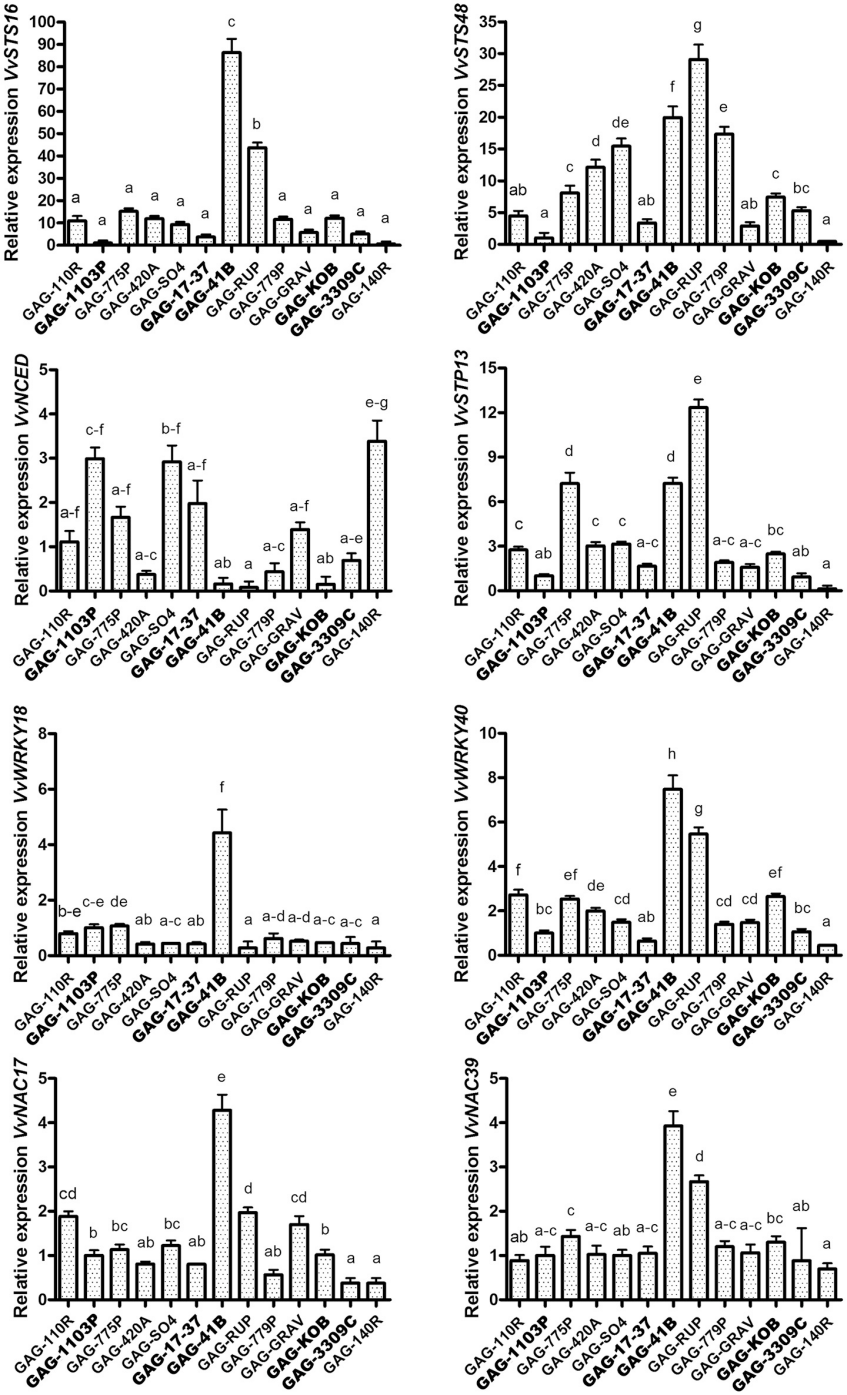
**Validation of the RNA sequencing results**. Relative expression levels obtained by RT-qPCR analysis of the *VvSTS16* (VIT_16s0100g00920), *VvSTS48* (VIT_16s0100g01200), *VvNCED* (VIT_02s0087g00930), *VvSTP13* (VIT_05s0020g03140), *VvWRKY18* (VIT_04s0008g05760), *VvWRKY40* (VIT_09s0018g00240), *VvNAC17* (VIT_19s0014g03290) and *VvNAC39* (VIT_07s0031g02610) genes tested in the 13 graft combinations. Lower case letters above bars denote significant differences by Tukey's HSD-test (*P* < 0.05). Data are expressed as mean ± SE (*n* = 3). The combinations chosen for RNA-seq analyses are indicated in bold font.

The rootstocks Kober 5BB, 3309 Couderc, 17–37 Mgt, and 41 B Mgt induced the activation of phenylpropanoid metabolism in “Gaglioppo” (Cluster 1) at very high levels in respect to 1103 Paulsen (e.g., through the up-regulation of about 40 genes coding for stilbene synthases). In particular, the 41 B Mgt rootstock induced high activation of these transcripts, with many stilbene synthase genes showing up to 200-fold up-regulation (Figure 3C). We selected two stilbene synthases (*VvSTS16*-VIT_16s0100g00920 and *VvSTS48*-VIT_16s0100g01200), members of two different sub-groups of *STS* (Vannozzi et al., 2012), with different transcription induction in response to stresses. *VvSTS48* is a gene induced in particular after downy mildew infection, while *VvSTS16* is activated in response to UV-C stress (Vannozzi et al., 2012). We extended the qRT-PCR analyses to all graft combinations. In addition, to validate the RNA-seq data (Supplementary Figure S3), we observed a strong activation of *VvSTS16* and *VvSTS48* in “Gaglioppo” grafted onto 41 B Mgt and Rupestris du Lot. Conversely, 1103 Paulsen and 140 Ruggeri induced transcription levels up to 100 times lower in comparison to GAG-41B (Figure 4). In addition, GAG-420A, GAG-779P and GAG-SO4 also showed high levels of *VvSTS48* in leaves. A possible explanation for this marked activation of stilbene-related genes could be that an active photosynthetic process is usually coupled with active growth of the plant (Corso et al., 2015), whereas in this case, interestingly, despite the up-regulation of photosynthesis—related genes (Supplementary Figure S2B), GAG-41B showed very low productivity and vigor, as already discussed (Table 1). Taking into account that imbalance between light capture and its utilization can result in the production of ROS responsible for the oxidative damage of cellular components (Apel and Hirt, 2004; Kar, 2011), we hypothesize that this imbalance could induce the 41 B Mgt rootstock to up-regulate stilbene-related genes that, with their ROS-scavenging activity, can protect the plant from oxidative damage. Moreover, this indication could be corroborated by the fact that resveratrol is induced by reactive oxygen species such as H_2_O_2_ (Wang J. F. et al., 2015). In addition to *STSs*, other genes involved in the prevention of oxidative stress, such as peroxidases and glutathione S-transferases, were up regulated in particular in GAG-41B (Cluster 1, Supplementary Table S4).

**Figure 5:**
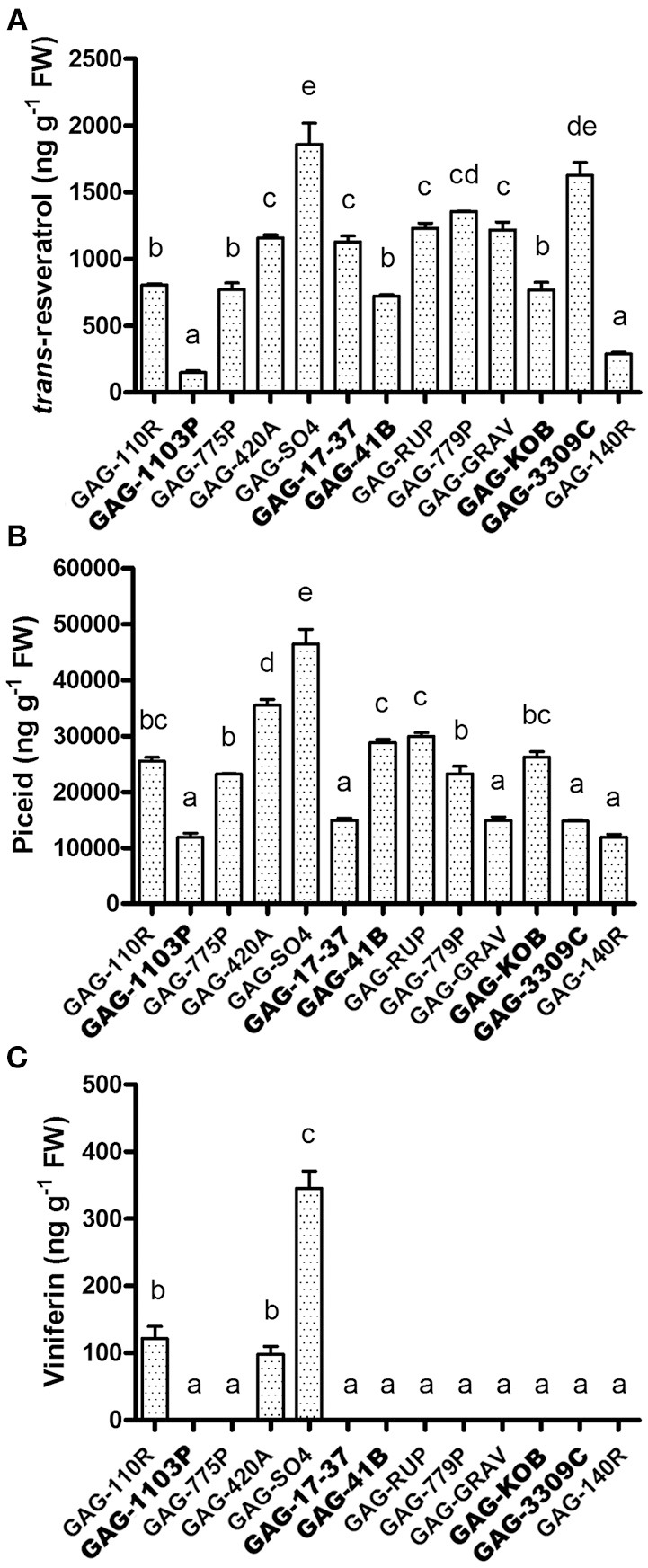
**Analysis of stilbenic compounds in scion leaves of 13 graft combinations**. Quantification of *trans*-resveratrol **(A)**, piceid **(B)** and viniferin **(C)** assessed in the scion leaves. Lower case letters above bars denote significant differences by Tukey's HSD-test (*P* < 0.05). Data are expressed as mean ± SD (*n* = 3). The combinations chosen for RNA-seq analyses are indicated in bold font.

The high level of *STS* transcription reported above in some graft combinations (Figure 4) suggested an increase in stilbene metabolite synthesis and accumulation in leaves. The quantification of resveratrol, viniferin, and piceid contents in leaves was performed in all 13 graft combinations available. *Trans*–resveratrol (*trans*-3,5,4′,-trihydroxy-*trans*-stilbene), its glucoside precursor *trans*-piceid (resveratrol-3-β-mono-D-glucoside) and the resveratrol dehydrodimer ε-viniferin were chosen since are low molecular weight phenolic compounds that act as antifungal or as phytoalexins common in grape (Bavaresco and Fregoni, 2001; Vincenzi et al., 2013). *Trans*-piceid was detectable in all samples, with a higher accumulation in GAG-420A and GAG-SO4 (Figure 5A). This last graft combination also showed a higher content of *trans*-resveratrol, together with GAG-3309C and, in general, *trans*-resveratrol showed a very similar trend of accumulation to *trans*-piceid (Figure 5B). Interestingly, not all the graft combinations produced a detectable level of viniferin, a more toxic derivative of *trans*-resveratrol (Figure 5C). This stilbenoid oligomer was detected only in GAG-110R, GAG-420A, and GAG-SO4.

#### ABA metabolism

It is well-known that ABA is involved in many plant-stress responses, and although this hormone has been extensively studied in terms of its function in plant drought tolerance, it plays a crucial role also in response to biotic stresses (Cao et al., 2011; Spence and Bais, 2015). ABA modulates the defense against the pathogen through different ways, e.g., interfering with other hormones such as salicylic acid or with ROS accumulation (Asselbergh et al., 2008).

**Figure 6:**
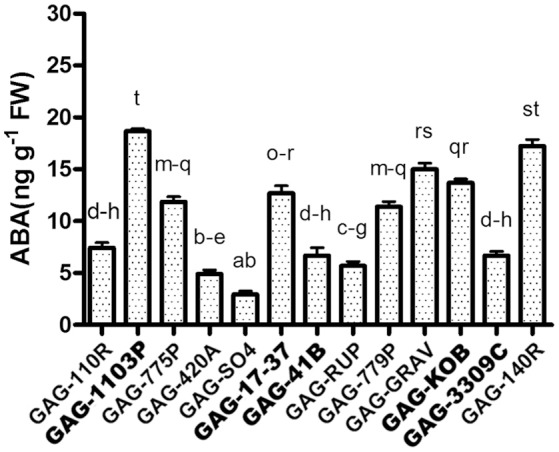
**Analysis of endogenous ABA levels**. Measurement of the leaves active ABA content in the 13 graft combinations. Lower case letters above bars denote significant differences attested by Tukey's HSD-test (*P* < 0.05). Data are expressed as mean ± SE (*n* = 3). The combinations chosen for RNA-seq analyses are indicated in bold font.

Our transcriptomic results showed an interesting up-regulation of ABA metabolism—related genes peculiar of GAG-1103P. In order to gain information at the metabolite level and to investigate further the rootstock/scion interaction, we performed the quantification of the foliar ABA content in all 13 available graft combinations (Figure 6). Higher ABA levels were measured in GAG-140R and GAG-1103P, confirming what emerged from the RNA sequencing data, whereas the combinations GAG-SO4, GAG-420A, GAG-RUP, GAG-3309C, GAG-110R, and GAG-41B showed a lower foliar ABA content. These results show how the rootstocks induced different ABA level fluctuations in the scion “Gaglioppo.” In addition, we monitored the leaf expression levels of a gene involved in ABA biosynthesis, i.e., 9-cis-epoxycarotenoid dioxygenase (*VvNCED*, VIT_02s0087g00930; Figure 4). The *VvNCED* transcript showed a similar trend in respect to the metabolite level, with the exception of some combinations, in particular GAG-SO4. The importance of ABA in root-to-shoot signaling and in the stomatal regulation is well-established (Heilmeier et al., 2007; Lovisolo et al., 2008; Tombesi et al., 2015), and it is well-known that the leaf hormone level is determined not only by biosynthesis but also by ABA transported from the root (Zhang et al., 1987; Lovisolo et al., 2016). This could explain the discrepancies between *VvNCED* expression and the ABA level in some combinations and underlines the importance of the rootstock influence. Very recent studies have added a further level of complexity to ABA signaling in plants, showing that leaf biosynthesized ABA is transported via phloem to the roots where it controls root architecture and growth by competing with IAA metabolism (Manzi et al., 2015; McAdam et al., 2016). Since we found different *VvNCED* expression levels in the scion “Gaglioppo,” depending on the graft combination, we can suggest that the rootstock controls leaf ABA levels, not only by providing ABA from the roots via the xylem, but also by directly affecting ABA biosynthesis in the leaf that can be translocated via the phloem to the roots.

#### Carbohydrate metabolism

In terms of carbohydrate metabolism, sugars involved in priming plant defense responses against fungal pathogens have been reported for both mono- and dicotyledons. Gòmez-Ariza et al. (2007) demonstrated the sucrose-mediated responsiveness of defense-related genes in rice and a subsequent improved resistance to *Magnaporthe oryzae* infection. Similarly, Lemonnier et al. (2014) demonstrated in *Arabidopsis* the key role of sugar transporter 13 (STP13) in intracellular glucose uptake. Here, a high constitutive level of STP13 led to an enhanced capacity to absorb glucose and to increase the basal resistance to *Botrytis cinerea* by limiting disease severity. Interestingly, *VvSTP13*, the ortholog of the *Arabidopsis* sugar transporter protein AtSTP13, was strongly up-regulated, particularly in GAG-41B leaves (Supplementary Table S4). In addition, qRT-PCR analysis of *VvSTP13* performed on all 13 graft combinations reported an up-regulation also in GAG-775P and GAG-RUP, whilst GAG-1103P, GAG-3309C, and GAG-140R showed significantly lower activation (Figure 4).

#### Stress-responsive transcripts

A large number of disease resistance protein genes belonging to the nucleotide-binding domain/leucine-rich repeat (NBS-LRR) class and several pathogenesis-related proteins (PRPs) were found to be up-regulated, particularly in GAG-41B, and down-regulated in GAG-1103P (Supplementary Table S4, Figure 3C). NBS-LRR genes have been largely studied and characterized in *Arabidopsis* as immune receptors against pathogens, and their importance in the innate immune system in plants (Chae et al., 2016) has posed new questions regarding the role(s) of the grapevine rootstock in how the scion perceives molecules released by pathogens; this requires further studies. Taken together, these results suggest the presence of pathogen-responsive priming genes mediated by sugar signaling, as previously reported in rice and tobacco (Murillo et al., 2003; Gòmez-Ariza et al., 2007). In addition, looking at the lipid metabolism category, several lipase and lipoxygenase isoform (*LOXs*) transcripts were more highly expressed in Cluster 1 (Supplementary Table S4). *LOX* isoforms encode enzymes that participate in the synthesis of jasmonic acid and have been widely associated with responses to biotic and abiotic factors, suggesting their involvement in priming defense mechanisms (Cervantes-Gámez et al., 2016; Chitarra et al., 2016). In the transcription factor activity regulation category, 27 *VvWRKY* genes were found to be significantly activated in Cluster 1, while only one was activated in Cluster 2 (Supplementary Table S4, Figure 3C). WRKY proteins are transcription factors that have been found to be involved in the control of a broad range of physiological processes and responses to biotic and abiotic stresses (Wang M. et al., 2015) and are organized in a large superfamily (59 genes) in *V. vinifera* (Wang et al., 2014). In our transcriptomic data, we found that about 50% of the confirmed *VvWRKY* genes in grapevine were activated, particularly *VvWRKY18* (VIT_04s0008g05760) and *VvWRKY40* (VIT_09s0018g00240) which were selected for RT-qPCR experiments involving all graft combinations (Figure 4). High expression of *VvWRKY18* was found only in GAG-41B; *VvWRKY40* was strongly expressed in GAG-41B and GAG-RUP, whereas it was repressed mainly in GAG-140R with regulation similar to that of *VvSTS16* (Figure 4). These genes belong to Group II-a of the phylogenetic tree of WRKY domains in both grapevine and *Arabidopsis* and are associated with abiotic and biotic stresses, probably in controlling basal defense responses (Wang et al., 2014). Although in the last decade considerable effort has been made in functional studies of WRKYs in physiological and plant defense processes, further studies are needed to clarify these mechanisms in the complex rootstock/scion interaction. *VvNAC* genes, another transcription factor family playing an important role in the response to stresses, were found to be significantly activated in Cluster 1 (17 genes up-regulated, in particular in GAG-41B Supplementary Table S4, Figure 3C). Overexpression of *NAC* improves pathogen tolerance in transgenic *Arabidopsis* (Puranik et al., 2012) and in our experiments we observed a strong up-regulation of *VvNAC17*—VIT_19s0014g03290 and *VvNAC39*—VIT_07s0031g02610 (Wang et al., 2013) in GAG-41B and partially in GAG-RUP (Figure 4).

### Could constitutive stilbenoid and ABA compounds trigger plant immunity responses? a preliminary indication

It is well-known how *Plasmopara viticola* and fungal infections in general induce the accumulation of stilbenes as a defense reaction, since these phytoalexins are biologically active compounds with antifungal activities (Jeandet et al., 2002; Chalal et al., 2014). Viniferin is highly toxic and can be considered an important marker for grapevine resistance to downy mildew (Pezet et al., 2004), but very little information is available on the role of constitutive stilbenoids (Duan et al., 2015).

**Figure 7:**
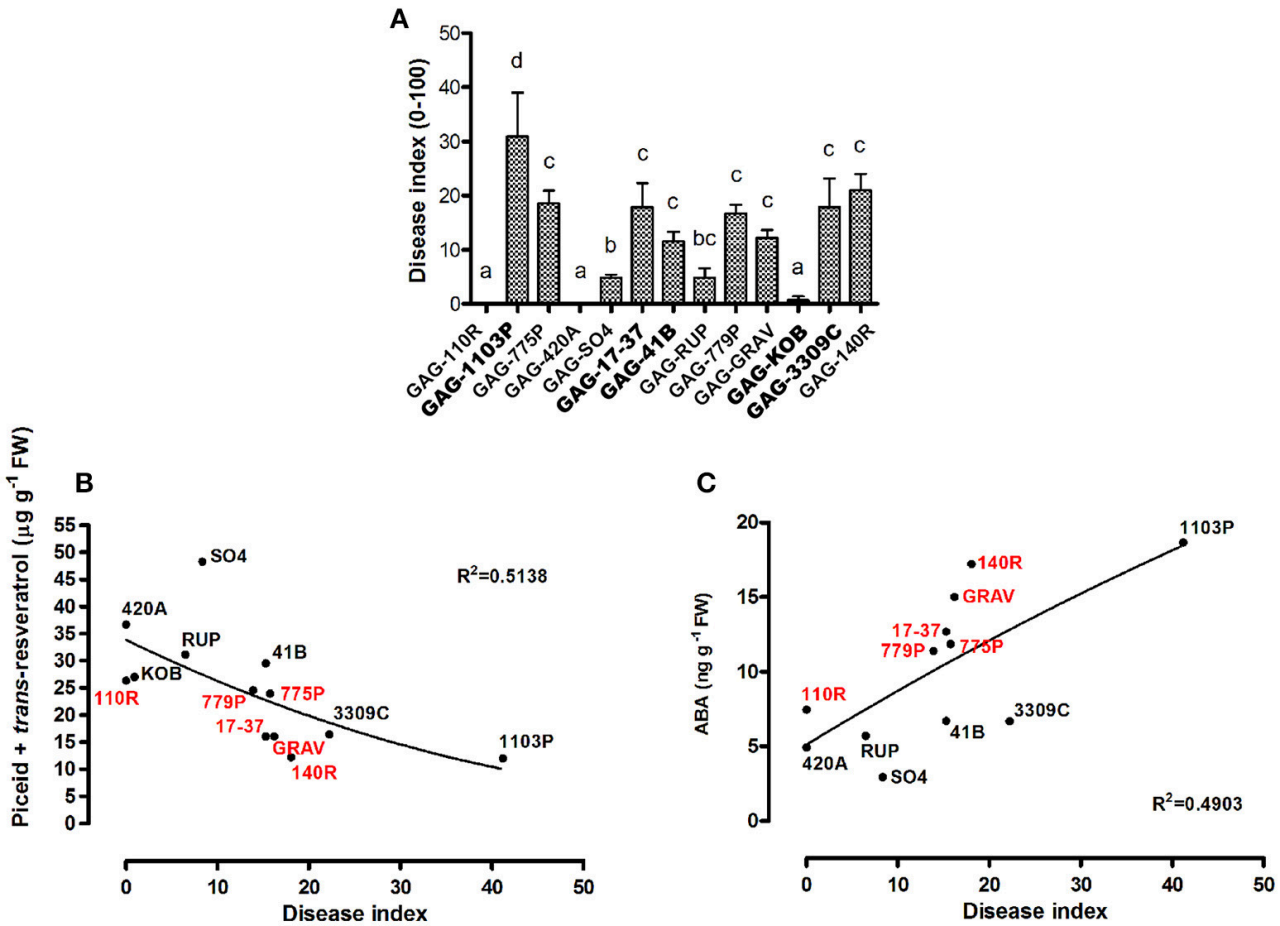
**Evaluation of disease resistance of scion leaves of 13 graft combinations against ***Plasmopara viticola*****. The Disease Index (DI) is expressed as the percentage of infected leaves. Lower case letters above bars denote significant differences by Tukey's HSD-test (*P* < 0.05). Data are expressed as mean ± SD (*n* = 3) **(A)**. The correlations between the disease index and the amount of piceid and trans-resveratrol (*p* < 0.05) **(B)** and between the disease index and the ABA content (*p* < 0.05) **(C)** related to each graft combination are plotted. Red and black labels are used to underline how graft combinations group according to the negative **(B)** or positive **(C)** correlation. The combinations chosen for RNA-seq analyses are indicated in bold font.

The results discussed above suggest a rootstock-dependent involvement of biotic stress responses. We wondered if this scenario and in particular the constitutive presence of stilbenoid and ABA compounds could influence *P. viticola* infection. For this purpose, leaf disks from “Gaglioppo” grafted onto different rootstocks were subjected to *P. viticola in vitro* infection (Figure 7A). Leaf disks were rated as resistant (disease index: 0–10%), moderately susceptible (11 to 30%), and susceptible (>30%). GAG-KOB, GAG-RUP, GAG-SO4, GAG-420A, and GAG-110R leaf disks were resistant to downy mildew and remained fresh and green during the evaluation period, suggesting in three of five combinations that the constitutive presence of viniferin (Figure 5C) could help counteract this pathogen (Figure 7A). The most susceptible combination was GAG-1103P, characterized by low levels of all tested stilbenoids, by the down-regulation of several transcripts involved in defense responses (Cluster 1, Supplementary Table S4) and by higher ABA content at the transcript and metabolite level, while the remaining samples were rated as moderately susceptible. Besides the role of viniferin, a negative correlation between the amount of *trans*-resveratrol and *trans*-piceid and the disease index of all samples was observed (Figure 7B), suggesting that graft combinations with a high level of *trans*-resveratrol and its storage form *trans*-piceid could be more prepared to respond to pathogen attack. While an inverse relationship between preformed flavonoids and the susceptibility of grapevines to downy mildew has been demonstrated (Agati et al., 2008; Latouche et al., 2013), in this work, we suggest a similar role for the preformed non-oxidized form of stilbenes. Resveratrol and its glycosylated form *trans*-piceid are produced in susceptible grapevines as response to infection of downy mildew (Pezet et al., 2004; Houillé et al., 2015), with limited antifungal activity when the infection is in progress. Our results suggest how these compounds, probably in association with other defense responses, could play an important role if present in a constitutive form before infection, likely because, as suggested by Pool et al. (1981), the availability and the speed of resveratrol synthesis are positively correlated with the resistance of grapevine varieties to fungal diseases. It is known moreover that pathogens can delay the expression of host genes belonging to the phenylpropanoid pathway during infection (Milcevicova et al., 2010), meaning that a primed state of expression of those genes can help the plant to be more ready to the pathogen attack. Similar indications come from a transcriptomic study on “Gala” apple scion grafted on different rootstocks, showing that differences in scion fire blight susceptibility were associated with high expression of phenylpropanoid and biotic and abiotic stress responses induced by specific rootstocks in uninfected trees (Jensen et al., 2012).

On the contrary, an interesting positive correlation between ABA content and disease index was observed in 12 graft combinations out of 13 (Figure 7C). Several studies report a negative role of ABA in plant immunity (reported in Cao et al., 2011) and many specific examples have shown that elevated ABA content in plants is associated with increased susceptibility to various bacterial and fungal pathogens, while decreased ABA content resulted in increased resistance (Spence and Bais, 2015), as observed in our results.

These findings open new perspectives about the growth-defense trade-offs in grapevine also in term of appropriate rootstock selection for viticulturists. It is well-established that growth-defense trade-offs have important consequences in physiological and carbon allocation costs shifting a pool of energy reserves toward growth or defense machineries (Messina et al., 2002; Huot et al., 2014). In general, several studies showed that plants resist against biotic attack producing defense compounds as innate immune responses (e.g., tannins and/or activating phenylpropanoid pathways), often associated with reduced growth (Bandau et al., 2015; Decker et al., 2016) as observed here in GAG-41B graft combination.

To date, the molecular mechanisms involved in plant growth-defense trade-offs need to be elucidated, since studies focused mainly on hormone crosstalk (Huot et al., 2014). Only recently researchers posed more attention to the fine-tuning transcriptional processes behind this phenomenon. For example, Campos et al. (2016) through epistatic gene interactions demonstrated that *Arabidopsis* growth inhibition in response to a biotic factor is not caused only by allocation of photoassimilates toward defense but by activating a molecular network involving jasmonate signaling. Furthermore, it has been reported that *V. vinifera* cells grown *in vitro* respond to methyljasmonate with increase of phytoalexin production, piceid in particular (Krisa et al., 1999). Those results together suggest as jasmonate could play a key role in regulating the signal transduction toward growth or defense. Coherently, our results showed how the 41B rootstock induced higher levels of stilbenoid genes in the scion (Cluster 1; Supplementary Table S4), lower vigor (Table 1) and higher expression of jasmonate synthesis and signaling genes (Cluster 1; Supplementary Table S4), in respect to the other rootstocks considered in the RNA–seq analysis.

In summary, our study showed a transcriptional picture that could elucidate how several rootstocks drive the fitness costs, associated with constitutive defense, modulating stress related genes in scion leaves as well as other transcripts not directly related to the phenotype. Growers for decades selected grapevine rootstocks to achieve an enhanced primed state against biotic or abiotic factors, regulating their growth-defense trade-offs to optimize plant fitness in response to a particular environment.

## Conclusions

It is well-known that different rootstocks confer different yield and phenological features to the grapevine scion; however, the results obtained in this work further improve our knowledge of the molecular and metabolic changes involved in this interaction. Following the trade-offs fashion, GAG-41B emerged as a graft combination that responds in a very different way with respect to the others, in particular with respect to GAG-1103P, which showed the opposite behavior in terms of molecular responses; GAG-KOB, GAG-3309C, and GAG17-37 had responses similar to GAG-1103P, although less pronounced. The rootstock 41 B Mgt was able to induce a number of genes involved in early responses to biotic stresses (e.g., *VvPRs, VvNBS-LRRs, VvLOX*s, *VvNACs, VvWRKYs*), with particularly high activation of secondary metabolism genes (*VvSTSs*). For these reasons, “Gaglioppo” grafted onto rootstock 41 B Mgt appears to be constitutively more prepared to respond to pathogen attack, although it is less productive and vigorous, whereas 1103 Paulsen seems to not induce scion responses against biotic stresses. An indication in this direction also emerged from the leaf disk assay regarding *in vitro* infection with *P. viticola*, where GAG-41B was rated as moderately susceptible and GAG-1103P was very sensitive to *Plasmopara* attack.

More in general our results give a first indication about the relationship among constitutive stilbenoids and ABA levels and disease susceptibility to a fungal pathogen. Interestingly stilbenoids and ABA showed an opposite trend in respect to the disease susceptibility, identifying the susceptible graft combination as the one with less stilbenoids and higher ABA level. These results should be further verified under different environmental conditions, at different developmental stages and using other cultivars as scion, in order to further assess the mechanisms of the complex rootstock/scion interaction. The results obtained in the present study, although representing only one time point of the grapevine season, provide an overall picture of the genetic and metabolic modulation inducted by several rootstocks in grapevine. These findings may facilitate the selection of appropriate graft combinations in the field, depending on the environment conditions, and help to identify candidate genes for further functional studies or for cis-genic and genome editing purposes.

## Author contributions

WC and IP performed most of the molecular analysis, analyzed the data and wrote the manuscript; CA and AM performed and elaborated RNA-seq data; PB collaborated to the molecular analysis; DS elaborated agronomical data; GGi performed the pathogenicity test; IS performed the HPLC-MS/MS analysis; MG, FM, and MD critically revised the manuscript; GGa designed the experiments, contributed to data analysis and to the writing; all authors read and approved the manuscript.

## Funding

This research was funded by Regione Calabria in the framework of PSR 2007–2013, Misura 124. IP was financed by the Italian Ministry of University and Research, FIR project RBFR13GHC5: “The Epigenomic Plasticity of Grapevine in Genotype per Environment Interactions.”

### Conflict of interest statement

The authors declare that the research was conducted in the absence of any commercial or financial relationships that could be construed as a potential conflict of interest. The reviewer JG and handling Editor declared their shared affiliation, and the handling Editor states that the process nevertheless met the standards of a fair and objective review.
