# Supplementary material for: Grapevine Grafting: Scion Transcript Profiling and Defense-Related Metabolites Induced by Rootstocks
Source: Front Plant Sci. 2017 Apr 27;8:654. doi: 10.3389/fpls.2017.00654 (PMC5407058; doi:10.3389/fpls.2017.00654)
Supplement: Supplementary file 1 [file Table1.PDF]

Supplemental Table S1. Sequencing and mapping statistics.

Summary of the sequencing statistics for each sample and the final number of mapping reads after quality filtering.

name	average fragment size	GC content	Sequenced Fragments	Mapped Reads	Mapped Percentage
GAG-KOB_RepA	328	45	32023234	27868098	88.10%
GAG-KOB_RepB	319	45	39488809	34421981	88.20%
GAG-KOB_RepC	324	45	33805934	29591660	88.40%
GAG-1103P_RepA	323	45	33256161	29173498	88.80%
GAG-1103P_RepB	318	45	34143985	30073626	89.00%
GAG-1103P_RepC	328	45	32040476	28108278	88.60%
GAG-3309C_RepA	334	45	30960999	26913130	87.80%
GAG-3309C_RepB	310	45	34821896	30326138	87.90%
GAG-3309C_RepC	346	45	34537492	30075617	88.20%
GAG-41B_RepA	326	45	33557356	29268872	88.10%
GAG-41B_RepB	322	45	35030877	29997600	86.60%
GAG-41B_RepC	322	45	34993160	30288847	87.50%
GAG-17-37_RepA	326	45	32022511	27803701	87.70%
GAG-17-37_RepB	322	45	34092941	29547153	87.50%
GAG-17-37_RepC	324	45	24768137	21570817	87.90%
