# Supplementary material for: Grapevine Grafting: Scion Transcript Profiling and Defense-Related Metabolites Induced by Rootstocks
Source: Front Plant Sci. 2017 Apr 27;8:654. doi: 10.3389/fpls.2017.00654 (PMC5407058; doi:10.3389/fpls.2017.00654)
Supplement: Supplementary file 5 [file Table5.PDF]

Supplemental Table S5. List of the oligonucleotides used for RT-qPCR analyses.

Name	Primer sequence (5'-3')	Gene annotation	Gene ID	Reference
VvACT_F<br>*VvACT_R*	GCCCCTCGTCTGTGACAATG<br>CCTTGGCCGACCCACAATA	Actin	VIT_04s0044g00580	Gambino et al. 2012
VvUBI_F<br>*VvUBI_R*	TCTGAGGCTTCGTGGTGGTA<br>AGGCGTGCATAACATTTGCG	Ubiquitin	VIT_16s0098g01190	Gambino et al. 2012
VvSTP13_F<br>*VvSTP13_R*	GGGTACGGCAATGGATTCG<br>CCCTCCCCATACACCACTAATCT	Sugar transporter 13	VIT_05s0020g03140	This work
VvSTS16_F<br>*VvSTS16/22_R*	CTTTTGACCCAATTGGAATCAAC<br>TGACATGTTCCCATATTCACTTAG	Stilbene synthase 16	VIT_16s0100g00920	Vannozzi et al. 2012
VvSTS48_F<br>*VvSTS48_R*	CTTGAAGGGGGAAAATGCT<br>TTACTGCATTGAAGGGTAAACC	Stilbene synthase 48	VIT_16s0100g01200	Vannozzi et al. 2012
VvWRKY18_F<br>*VvWRKY18_R*	GCTGCCACTCCTGCTTCAG<br>TTACTGTTGGGCGTGGAGAGA	WRKY 18	VIT_04s0008g05760	This work
VvWRKY40_F<br>*VvWRKY40_R*	CAACCTACGAAGGCGAGCAT<br>GCACCTGATGTGGGCTCAAT	WRKY 40	VIT_09s0018g00240	This work
VvNAC17_F<br>*VvNAC17_R*	GTCATCGTCGTCCCACCTC<br>AAGAACCTGTCATCGATCTCC	NAC domain containing protein	VIT_19s0014g03290	Pantaleo et al. 2016*
VvNAC39_F<br>*VvNAC39_R*	ACCCTTTTGCCTCTCAAGCA<br>GTCCTGCCACGACGATGTTA	NAC domain containing protein	VIT_07s0031g02610	This work
VvNCED_F<br>*VvNCED_R*	GCCCCAACCCCCAGTTC<br>GCATGCCATCACCATCAAAG	9-cis-epoxycarotenoid dioxygenase	VIT_02s0087g00930	Gambino et al. 2012

* **Pantaleo V, Vitali M, Boccacci P, Miozzi L, Cuozzo D, Chitarra W, Mannini F, Lovisolo C, Gambino G** (2016) Novel functional microRNAs from virus-free and infected *Vitis vinifera* plants under water stress. Scientific Reports **6:** 20167.
