# Supplementary material for: Grapevine Grafting: Scion Transcript Profiling and Defense-Related Metabolites Induced by Rootstocks
Source: Front Plant Sci. 2017 Apr 27;8:654. doi: 10.3389/fpls.2017.00654 (PMC5407058; doi:10.3389/fpls.2017.00654)
Supplement: Supplementary file 6 [file Image1.PDF]

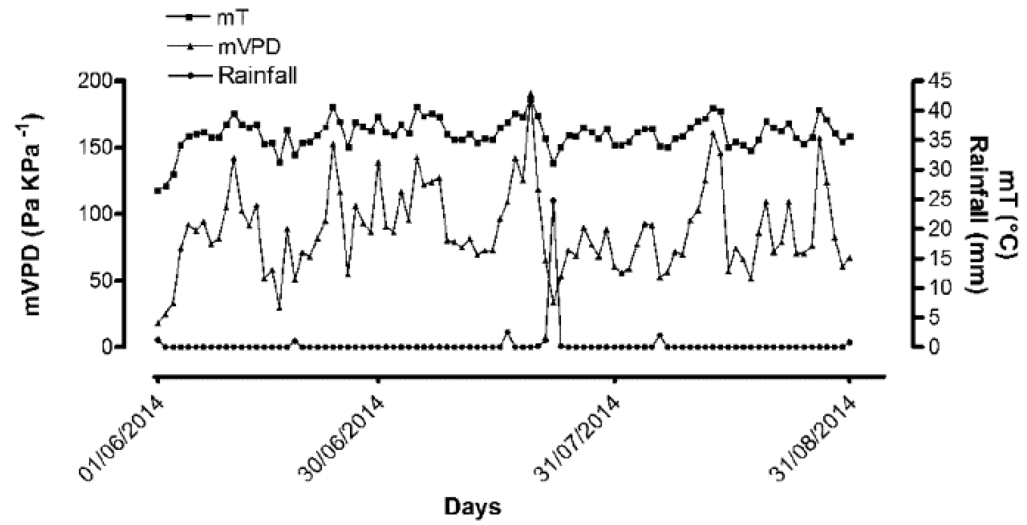

Supplementary Fig. S1.

Seasonal time course (2014) of maximum temperature (mT, square), midday vapor pressure deficit (mVPD, triangle), rainfall (circle).
