# Supplementary material for: Grapevine Grafting: Scion Transcript Profiling and Defense-Related Metabolites Induced by Rootstocks
Source: Front Plant Sci. 2017 Apr 27;8:654. doi: 10.3389/fpls.2017.00654 (PMC5407058; doi:10.3389/fpls.2017.00654)
Supplement: Supplementary file 7 [file Image2.PDF]

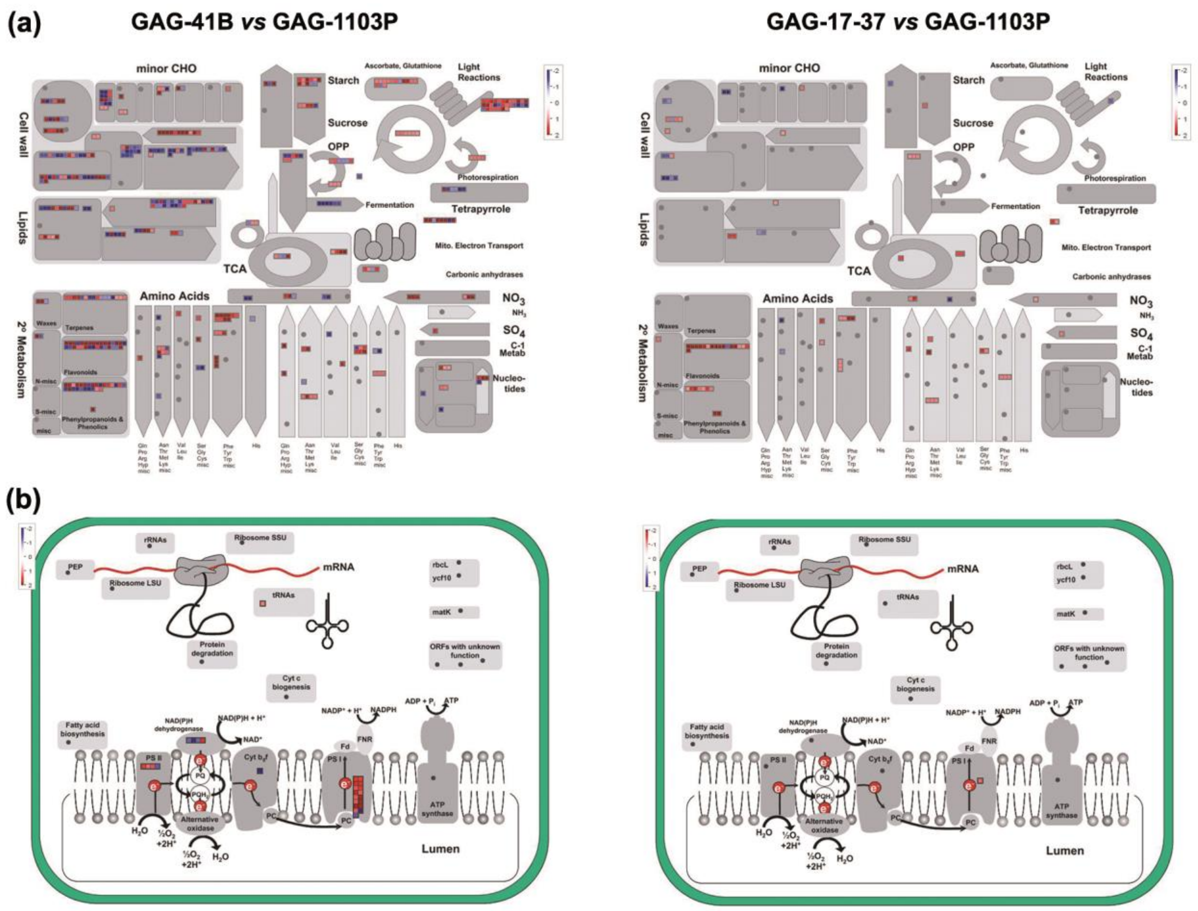

Supplementary Fig. S2. MapMan software was used to provide an overview of the effect of 41 B Mgt rootstock and 17-37 Mgt rootstock induced on the main metabolic pathways (a) and on the light-dependent reactions in the chloroplast (b). Log2 fold changes values referred to GAG-1103P are represented. Up-regulated and down-regulated transcripts are shown in red and blue, respectively.
