# Supplementary material for: Grapevine Grafting: Scion Transcript Profiling and Defense-Related Metabolites Induced by Rootstocks
Source: Front Plant Sci. 2017 Apr 27;8:654. doi: 10.3389/fpls.2017.00654 (PMC5407058; doi:10.3389/fpls.2017.00654)
Supplement: Supplementary file 8 [file Image3.PDF]

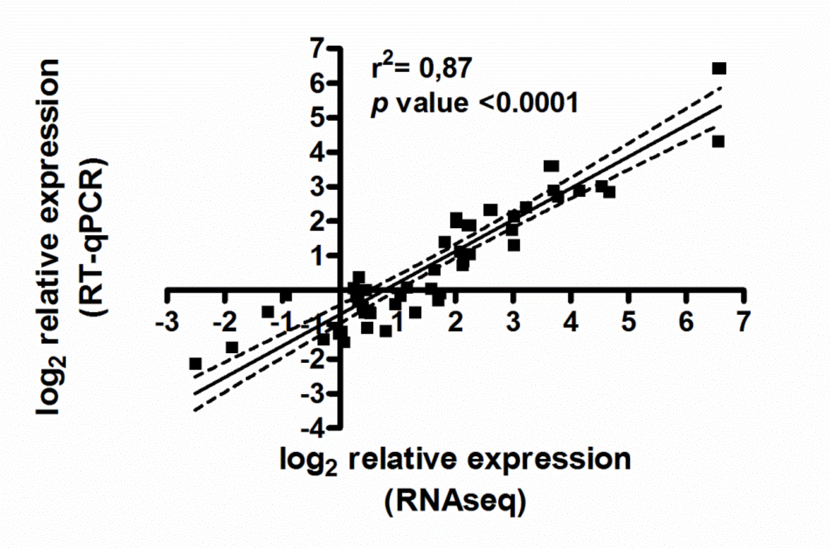

Supplementary Fig. S3. Correlation between log2 relative expression values measured by RT-qPCR and RNA sequencing analyses (black line, with 95% confidence interval).
